# S174. ABNORMAL SOCIAL COGNITION RELATED TO STRUCTURAL DISCONNECTIVITY IN SCHIZOPHRENIA

**DOI:** 10.1093/schbul/sby018.961

**Published:** 2018-04-01

**Authors:** Molina Vicente, Alba Lubeiro Juárez, Eva Sotelo, Mercedes Vaquero, Rodrigo de Luis-García

**Affiliations:** 1 University of Valladolid; 2 University Hospital of Valladolid

## Abstract

**Background:**

Social cognition impairments are found in schizophrenia patients and hamper their ability to form social relationships. The biological underpinnings of this social cognition impairment are poorly investigated. We hypothesize that structural disconnectivity, which is replicated in schizophrenia, might has a relevant role in social cognition.

**Methods:**

The study we present here is under development. We have assessed social cognition using the Mayer, Salovey and Caruso emotional intelligence test (MSCEIT) in 30 patients with schizophrenia and 20 healthy controls. Structural connectivity is assessed with anatomical and Diffusion weighted (DWI) images acquired in a 3 Tesla MRI system. Anatomical and DWI images are processed to obtain fractional anisotropy (FA) values in the tracts connecting prefrontal cortex with anterior cingulate, superior temporal gyrus, insula and superior parietal cortex. The following statistics are assessed i) the differences in MSCEIT scores between patients and controls, ii) the differences in FA values between groups, iii) the relation between MSCEIT punctuation and FA values.

**Results:**

In our preliminary analyses, patients show significantly lower MSCEIT scores. Furthermore, MSCEIT scores are directly related to FA values in the tracts connecting prefrontal cortex to anterior cingulate and superior temporal gyrus in the patients.

**Discussion:**

Social cognition impairments seem to be associated with altered structural connectivity in the patients.

